# Cryptotanshinone attenuates the stemness of non-small cell lung cancer cells via promoting TAZ translocation from nuclear to cytoplasm

**DOI:** 10.1186/s13020-020-00348-4

**Published:** 2020-06-30

**Authors:** Linling Jin, Zhenzhen Wu, Yanli Wang, Xin Zhao

**Affiliations:** Department of Respiratory & Critical Care Medicine, Jiangsu Province Hospital, The First Affiliated Hospital of Nanjing Medical University, 300 Guangzhou Rd, Nanjing, 210029 People’s Republic of China

**Keywords:** Cryptotanshinone, Stemness, Non-small cell lung cancer, TAZ, Hippo

## Abstract

**Background:**

Cancer stem cells (CSCs) are regarded as the root of tumor progression, thus representing an anti-cancer therapy through targeting this cell sub-population.

**Methods:**

Non-small cell lung cancer (NSCLC) CSCs were enriched by non-adherent spheroid formation analysis. Lentivirus infection was used to stably change gene expression. Cell cycle, EdU incorporation, cell apoptosis, cell viability, ALDH1 activity, spheroid formation and in vivo tumor initiation assays were performed to detect the effects of Cryptotanshinone (CT), a traditional Chinese herb medicine, on the stemness of NSCLC cells. RNA-sequencing combined qRT-PCR and western blot analysis were constructed to explore the underlying mechanism contributing to CT-mediated effects.

**Results:**

CT could attenuate the stemness of NSCLC CSCs, as evident by the reduced spheroid formation ability, stemness marker expression and ALDH1 activity. Additionally, CT provoked NSCLC CSCs entry into the cell cycle. RNA-sequencing analysis showed that Hippo signaling pathway was highly enriched in NSCLC CSCs with CT treatment. Further experiments disclosed that CT decreased TAZ (a regulatory master of Hippo pathway) expression via promoting its nuclear-cytoplasm translocation in NSCLC CSCs. Also, overexpression of TAZ partially saved the attenuation of CT on the stemness of NSCLC CSCs. Notably, CT enhanced the sensitivity of tyrosine kinase inhibitor (TKI) and chemotherapy in NSCLC CSCs.

**Conclusions:**

This work reveals that CT attenuates NSCLC CSC stemness, implying the possibility of CT as an adjuvant therapy for NSCLC.

## Background

Lung cancer is common malignant tumor in clinic with a high morbidity and mortality and ranks the first in tumors, among which non-small cell lung cancer (NSCLC) accounts for 80% [1]. Because NSCLC has no obvious symptoms in the early stage, most of the patients are diagnosed with advanced stage. At present, radiotherapy, chemotherapy and targeted drug therapy are the main treatment in clinic, however, many patients, such as KRAS-mutant NSCLC, are not response or resistant to chemotherapy and targeted drug therapy [2]. Therefore, it is important to find novel ways to treat NSCLC.

Tumor initiating cells, also known as cancer stem cells (CSCs), have been regarded as the root of tumorigenesis [3]. It was found that targeting CSCs or attenuating the stemness could suppress tumor development, progression and decrease drug resistance. For example, salinomycin kills breast CSCs by sequestering iron in lysosomes and thus arrest tumor progression [4, 5]. Aspirin attenuates the chemoresistance of breast cancer cells by suppressing signaling necessary for CSC progression [6]. Resveratrol exerts its anti-tumor effects in breast cancer via inducing the apoptosis of CSCs through modulating fatty acid synthase expression [7]. Cryptotanshinone (CT) is the main lipid-soluble component and one of the many monomers of Salvia miltiorrhiza [8]. CT has been shown to harbor many pharmacological activities, such as anti-cholinesterase, anti-inflammatory, anti-oxidation, anti-bacterial, anti-tumor and anti-platelet aggregation [9]. Recent study has shown that as the main components of Salvia miltiorrhiza, CT, tanshinone I dihydrotanshinone and tanshinone IIA are the ideal inhibitors of P-gp [10], which is overexpressed in CSCs [11]. CT and tanshinone IIA are both the lipid-soluble constituents of Salvia miltiorrhiza, notably, tanshinone IIA has been shown to reduce the stemness of cervix carcinoma cells [12], glioma stem cells [13] and breast cancer stem cells [14]. A previous study has shown that CT could reduce the spheroid formation capacity and downregulate the expression of stemness regulators in prostate cells and prostate CSCs [15]. Additionally, CT enhances paclitaxel sensitivity of tongue squamous cell carcinoma through suppressing the JAK/STAT3 pathway [16]. These results imply that CT may hold the similar effects in regulating the stemness of tumor cells as tanshinone IIA.

In the current work, NSCLC CSCs were enriched and collected by spheroid formation analysis, and subjected to experiments. We found that CT reduced the stemness of NSCLC CSCs in a concentration-dependent manner. Additionally, CT provoked the NSCLC CSCs into cell cycle. The mechanistic studies showed that CT activated the Hippo pathway, as evident by increasing the nuclear-cytoplasm translocation of TAZ. Finally, it was found that CT sensitized NSCLC CSCs to chemotherapy and tyrosine kinase inhibitor (TKI) treatment. These results suggest that CT could be used as a combination with TKI or chemotherapy for NSCLC patients.

## Materials and methods

### Cell cultures and reagents

Human NSCLC cell lines A549 and H1299 were purchased from Cobioer (Shanghai, China). Cells were maintained in 1640 medium (Hyclone, South Logan, UT) containing 1% streptomycin and penicillin as well as 10% fetal bovine serum (Hyclone), and cells were cultured at 37 °C under humidified air with 5% CO_2_. Cell lines have been tested and authenticated by short tandem repeat (STR) DNA profiling method. Cisplatin and Erlotinib were purchased from Selleck Chemicals (Houston, TX, USA).

### Quantitative real-time PCR (qRT-PCR)

Total RNA was extracted using TRIzol™ Reagent (Thermo Fisher Scientific, Waltham, MA, USA) and cDNA was reversely synthesized with High-Capacity cDNA Reverse Transcription Kit with RNase Inhibitor (Thermo Fisher Scientific). mRNA expression levels were measured using Rapid SYBR^®^ Green qPCR kit (Sigma, St.Louis, MO) on on the Illumina Eco™ Real-Time PCR System. GAPDH expression was used as an endogenous reference. 2^−△△ct^ method was performed to measure the relative expression levels.

### RNA sequencing analysis

RNA-sequencing analysis was constructed by Genedenovo (Guangzhou, China).

### Western blot analysis

The detailed procedure was mentioned in the previous work [17]. The original images of western blots were denoted in Additional file 1: Figure S1.

### Lentivirus vector construction

TAZ overexpression lentivirus, knockdown lentivirus and control vectors were constructed by Biomics (Nantong, China), denoted as Len-TAZ and Len-TAZ-kd.

### Cell cycle assay

Cell cycle analysis was performed with the cell cycle assay kit (Yifeixue, Nanjing, China) following the recommendation procedures on flow cytometry.

### 5-Ethynyl-2′-deoxyuridine (EdU) incorporation assay

EdU incorporation assay was performed to measure the speed of DNA synthesis with a BeyoClick™ EdU-647 kit (Beyotime, Beijing, China).

### Cell apoptosis assay

Annexin V-FITC/PI Apoptosis Detection Kit (Meilune, China) was used to detect cell apoptosis.

### Cell viability assay

Cells were digested and seeded into 96-well plates at 4000 cells/well, after 12 h, cells were treated drug for 48 h. Then cell viability was measured using Cell Counting Kit-8 (Meilunbio, Dalian, China).

### Spheroid formation analysis

The process is referred to the previous study [18]. Briefly, NSCLC cells were maintained in DMEM-F12 medium containing B27 (Sigma, 20 ng/ml) and EGF (BD Biosciences, San Jose, CA, 10 ng/ml) in non-adherent 24-well plates (Corning, NY) at 1000 cells/well for 10 days. After then, spheroids with more than 50 μm were photographed and counted. For analysis on spheroids, spheroids were collected, trypsinized and re-seeded in plates. The spheroid-derived cells were incubated until the end of each experiment, and fresh spheroids were collected for each experiments.

### ALDH1 activity assay

Acetaldehyde dehydrogenase (ALDH) Assay Kit (Solarbio, Beijing, China) was used to determine ALDH1 activity in NSCLC cells according to the manufacturer’s protocols.

### In vivo tumor initiation assays

All animal experiments were performed with the approval of Ethics Committee for Animal Experimentation of Bengbu Medical College. 6–8 weeks of athymic BALB/c nude mice were purchased from the Gempharmatech (Nanjing, China). For analyzing of CT on the tumorigenic ability of NSCLC spheroids, spheroids were co-cultured with CT for 72 h before implanting in mice, and then subcutaneously implanted in mice with the same cell number. After 12 days, all mice were killed and tumor tissues were collected and weighed. The tumor-initiating ability was evaluated by calculating the tumor-formation rate. The CSC rate was calculated using ELDA software (http://bioinf.wehi.edu.au/software/elda/).

### Statistical analysis

Results were expressed as Mean ± SD and analyzed using Graphpad Prism (Version X; La Jolla, CA, USA). Student’s t test was used to assess the differences between groups. P < 0.05 was considered statistically significant.

## Results

### Non-adherent spheroids formed by NSCLC cells exhibit the CSCs-like traits

**Fig. 1:**
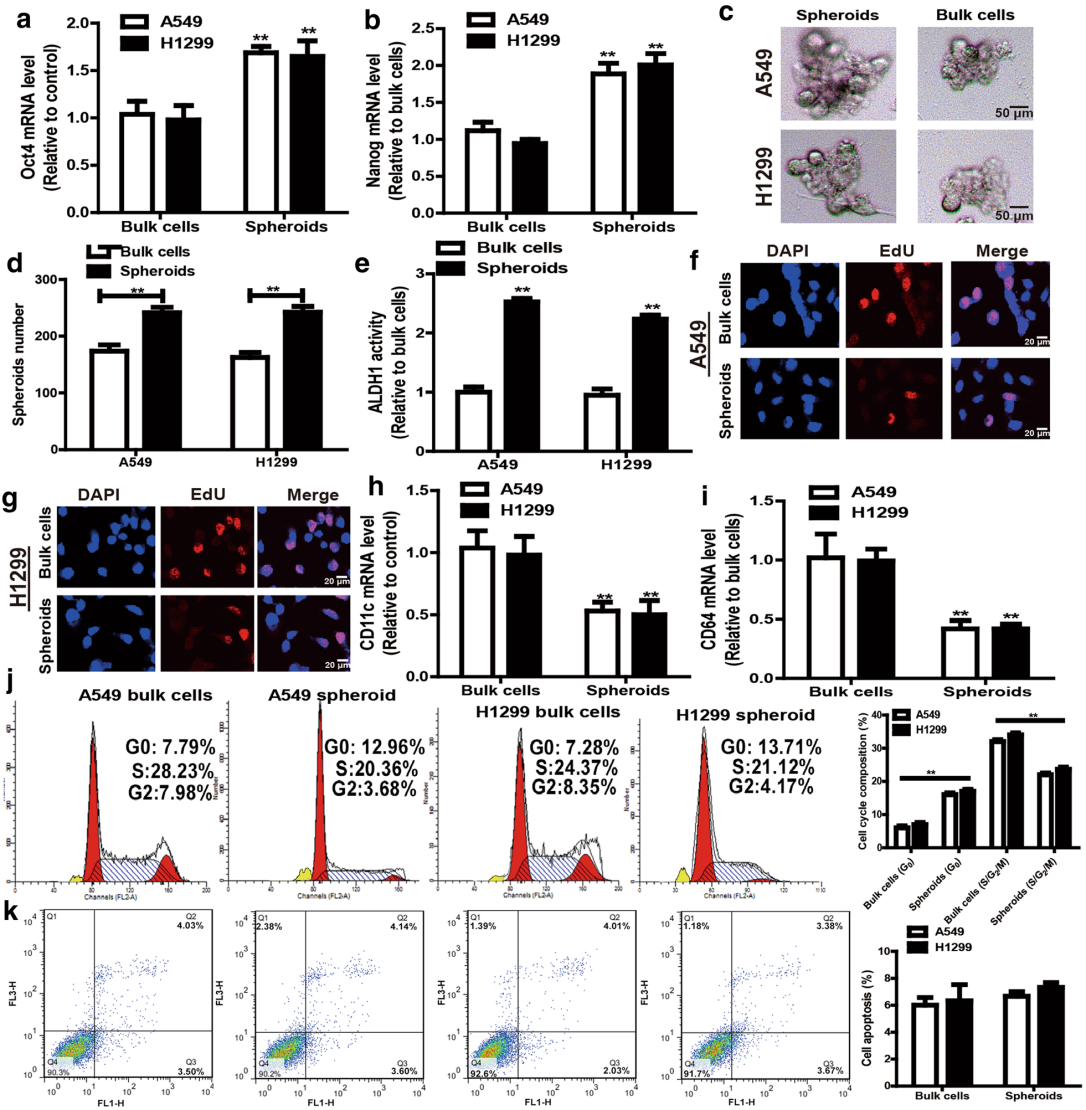
Non-adherent spheroids formed by NSCLC cells exhibited CSCs-like traits. **a**, **b** The expression levels of stemness markers (Oct4 and nanog) were detected in NSCLC cells and non-adherent spheroids. **c**, **d** The spheroid formation ability was determined in NSCLC spheroids and bulk cells via measuring spheroid size (**c**) and number (**d**). **e** ALDH1 activity was evaluated in NSCLC spheroids and bulk cells. **f**, **g** The EdU incorporation was evaluated in NSCLC spheroids and cells. **h**, **i** The mRNA levels of differentiation markers (CD11c and CD64) were examined in NSCLC spheroids and cells. **j** The cell cycle composition was determined in HCC spheroids and cells. **k** The cell apoptotic rate was measured in HCC spheroids and cells. Data were presented as the mean ± sd, **P < 0.01 vs. Bulk cells

Because non-adherent spheres have been shown to exhibit CSCs-like traits [19], NSCLC CSCs were enriched and collected via spheroid formation analysis. The CSCs-like traits were firstly identified through examining stemness marker expression, spheroid-forming ability and ALDH1 activity. As expected, the non-adherent spheroids harbored higher expression levels of stemness markers (oct4 and nanog), stronger capacity of spheroid formation and higher level of ALDH1 activity compared to adherent NSCLC cells (Fig. 1a–e). Since CSCs are always at a quiescent stage which helps them escape from being killed by drugs [20], we compared the cell cycle status between NSCLC cells and NSCLC non-adherent spheroids. It was found that NSCLC non-adherent spheroids exhibited a decreased EdU incorporation (Fig. 1f, g) and expression of differentiation genes (CD64 and CD11c) [21] (Fig. 1h, i). Additionally, NSCLC non-adherent spheroids held the remarkably upregulated ratio of quiescent (G_0_) cells and downregulated S/G_2_/M cells (Fig. 1j). Notably, cell apoptosis displayed no difference between NSCLC cells and non-adherent spheroids (Fig. 1k). Overall, these results indicate that the non-adherent NSCLC spheroids could be used as the model of NSCLC CSCs.

### CT attenuates the stemness of NSCLC CSCs

**Fig. 2:**
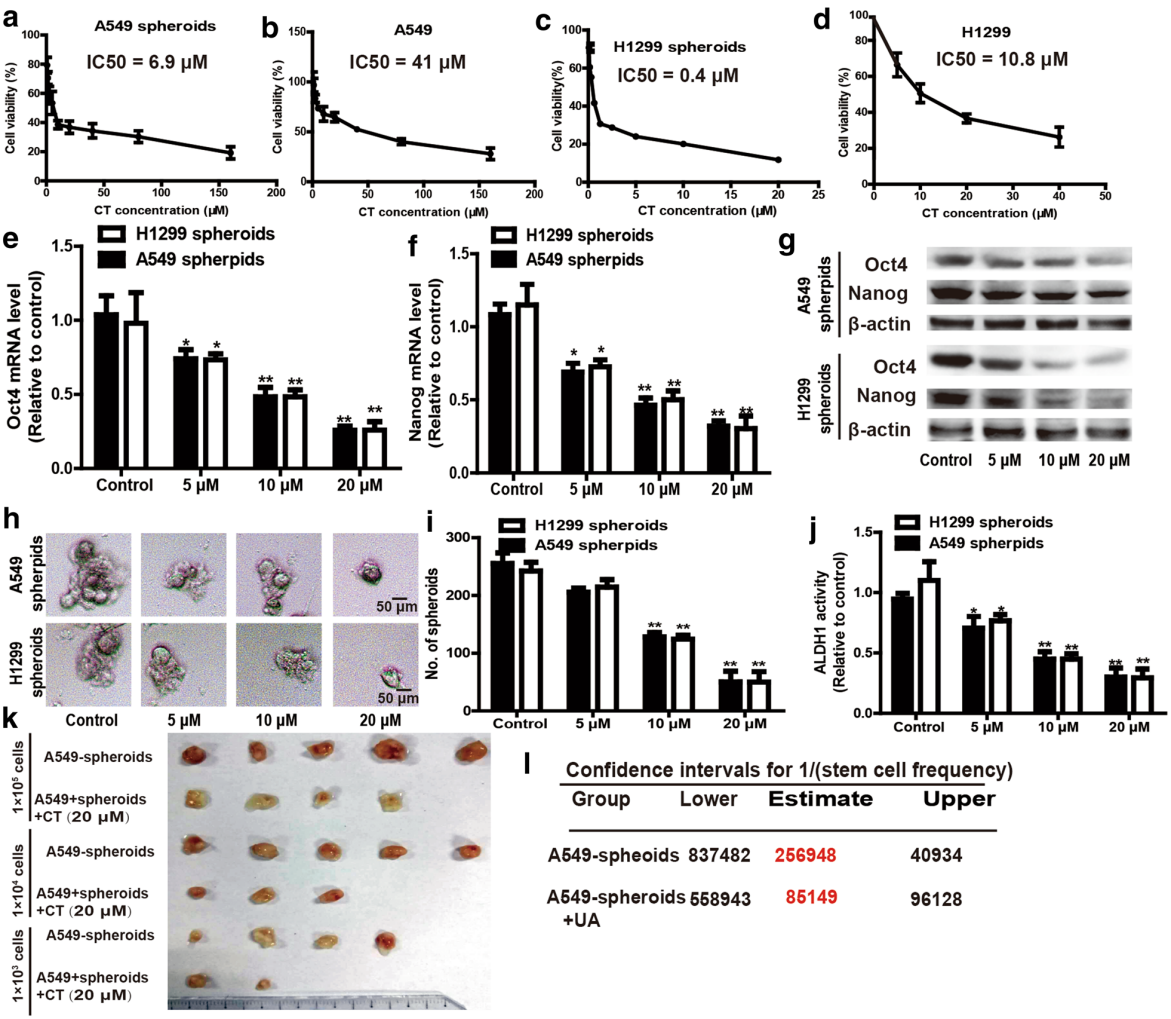
CT attenuates the stemness of NSCLC CSCs. **a**–**d** The IC50 values were measured in NSCLC cells and spheroids. **e**–**g** The mRNA levels of nanog and Oct4 were detected in the NSCLC spheroids treated with different concentrations of CT as indicated. **h**, **i** The capacity of spheroid formation was determined in the spheroids described in (**e**) via measuring spheroid size (**h**) and number (**i**). **j** ALDH1 activity was examined in the spheroids depicted in (**e**). **k**, **l** The tumor-initiation ability was evaluated in NSCLC CSC with or without CT treatment, and CSC rate was calculated using ELDA software. Data were presented as the mean ± sd, **P < 0.01 vs. control (Solvent control)

Following this, we investigated the CT effects on the stemness of NSCLC CSCs. As shown in Fig. 2a–d, CT displayed a stronger inhibitory effect on the viability of NSCLC CSCs than that in NSCLC cells, characterized as the lower IC50 values. Additionally, CT reduced the expression of stemness markers (oct4 and nanog) in a concentration-dependent manner (Fig. 2e–g). The spheroid-forming ability of NSCLC CSCs was attenuated by CT treatment, which is supported by the decreased spheroid number and size (Fig. 2h, i). CT decreased the activity of ALDH1 in NSCLC CSCs (Fig. 2j). Furthermore, the tumor-initiation ability of NSCLC CSCs was significantly attenuated by CT treatment, which is characterized by the decrease of tumor-formation rate and CSC rate (Fig. 2k, l).

### CT assists the NSCLC CSCs enter cell cycle

**Fig. 3:**
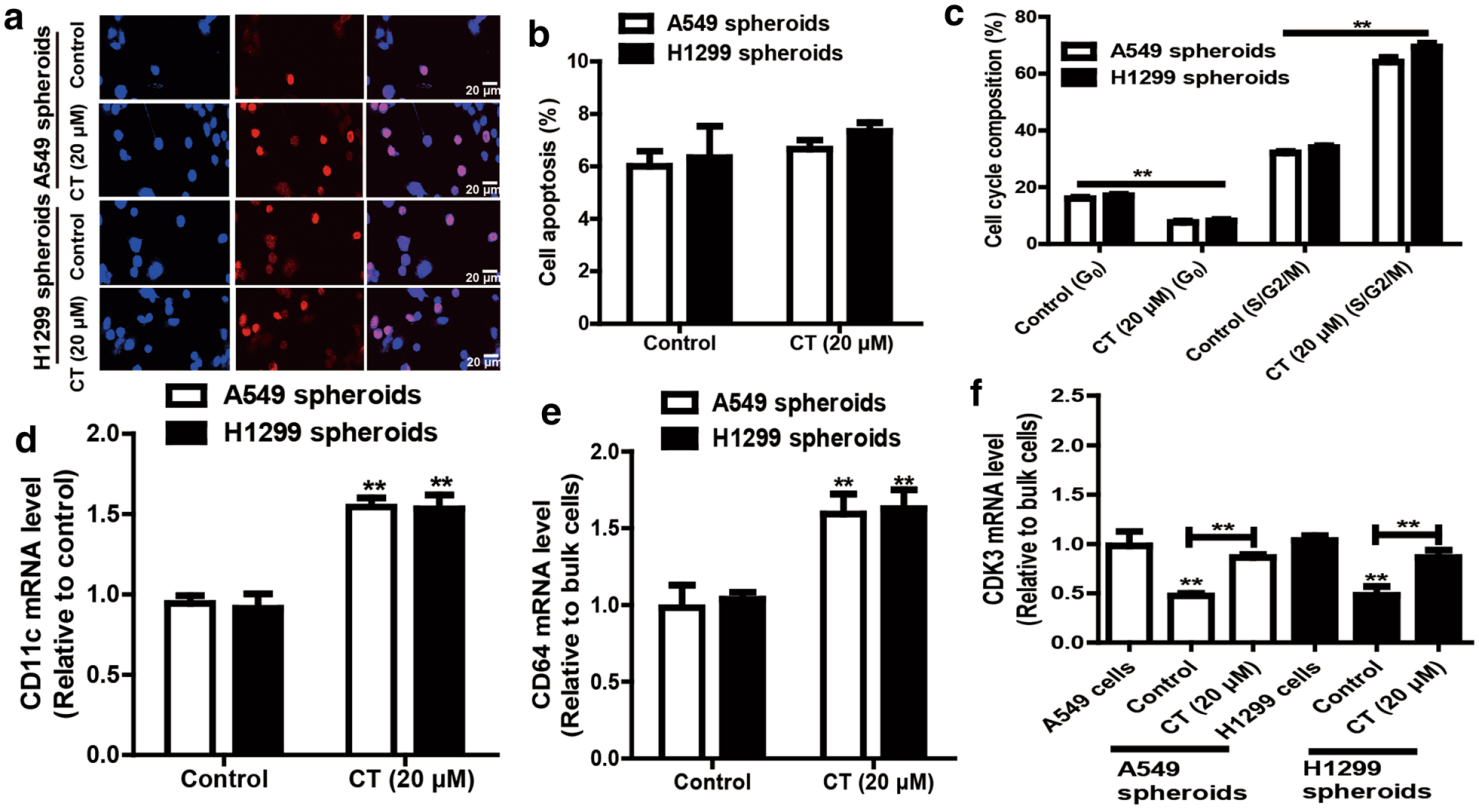
CT assists the NSCLC CSCs enter cell cycle. **a** EdU incorporation was evaluated in NSCLC spheroids treated with or without CT. **b** Cell apoptosis was examined in the spheroids described in (**a**). **c** Cell cycle composition was determined in the spheroids depicted in (**a**). **d**, **e** The mRNA levels of differentiation markers (CD11c and CD64) were detected in the spheroids described in (**a**). **f** CDK3 mRNA level was measured in NSCLC cells and spheroids treated with or without CT. Data were presented as the mean ± sd, **P < 0.01 vs. control (solvent control)

To further confirm the CT effects on the stemness of NSCLC CSCs, the differentiation, proliferative and clonogenic capacity was evaluated. As shown in Fig. 3a, b, NSCLC CSCs treated with CT displayed an increased EdU incorporation compared with control group, whereas the apoptosis of NSCLC CSCs was unaffected. Furthermore, the reduced ratios of quiescent (G_0_) cells and an increased S/G_2_/M cells were observed in NSCLC CSCs with CT treatment (Fig. 3c). Additionally, the expression of differentiation genes (CD64 and CD11c) was increased by CT in NSCLC CSCs (Fig. 3d, e). Since CDK3 is essential for G_0_ phase exit in cell cycle, CDK3 expression was determined and results indicated that CT increased the expression CDK3 in NSCLC CSCs (Fig. 3f). As a result, these results demonstrate that CT suppresses the stemness of NSCLC CSCs.

### Hippo pathway is activated in NSCLC CSCs with CT treatment

**Fig. 4:**
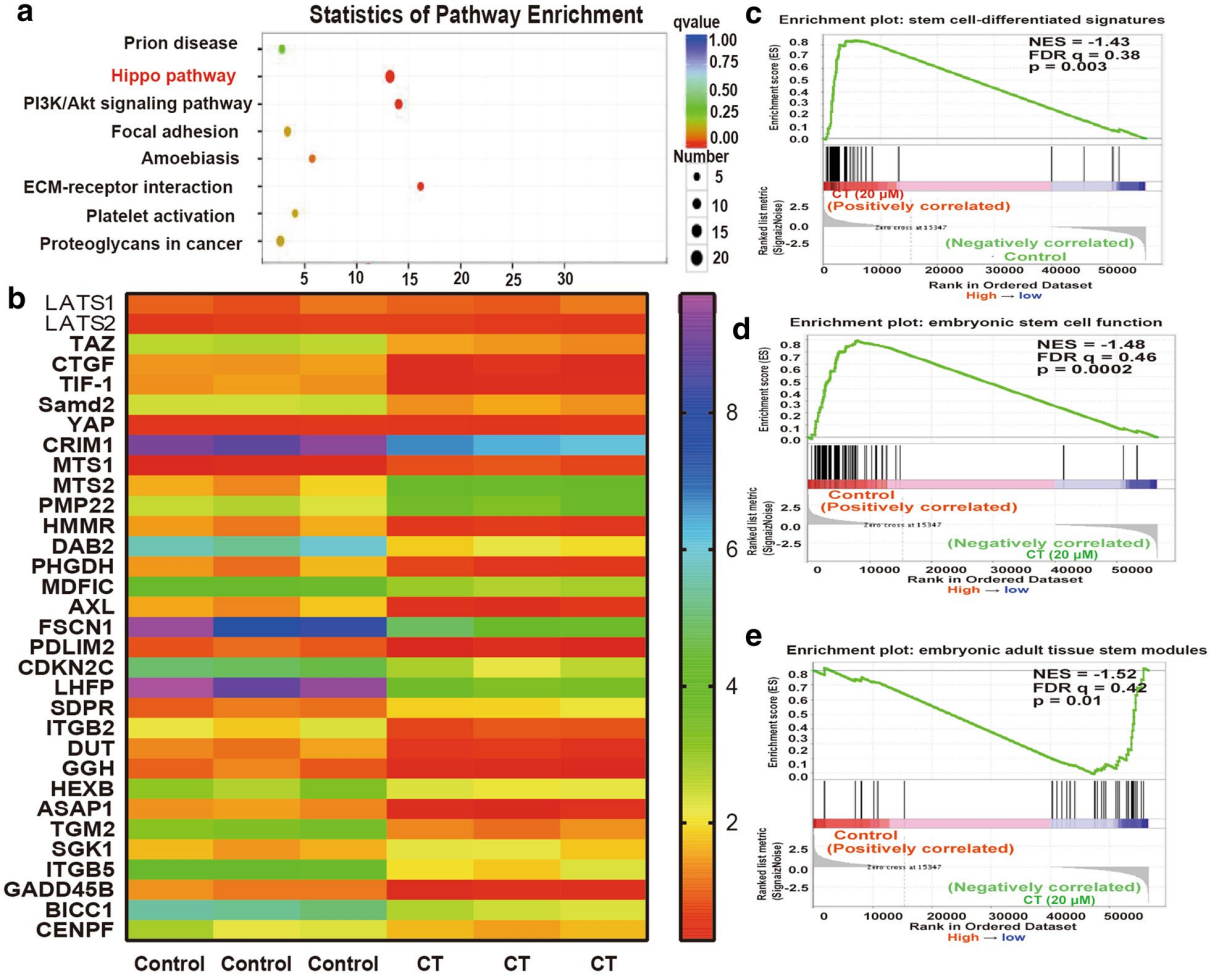
Hippo pathway is activated in NSCLC CSCs with CT treatment. (A) KEGG pathway analysis based on RNA-sequencing dataset showed that Hippo pathway was the most significantly enriched pathway in NSCLC spheroids with CT treatment. (B) The expression of Hippo signaling-related molecules was examined in NSCLC spheroids with or without CT treatment. (F - H) GSEA analysis was performed to analyze the enrichment of stem cell-differentiated signatures (F), embryonic stem cell function (G) and embryonic adult tissue stem modules (H)

**Fig. 5:**
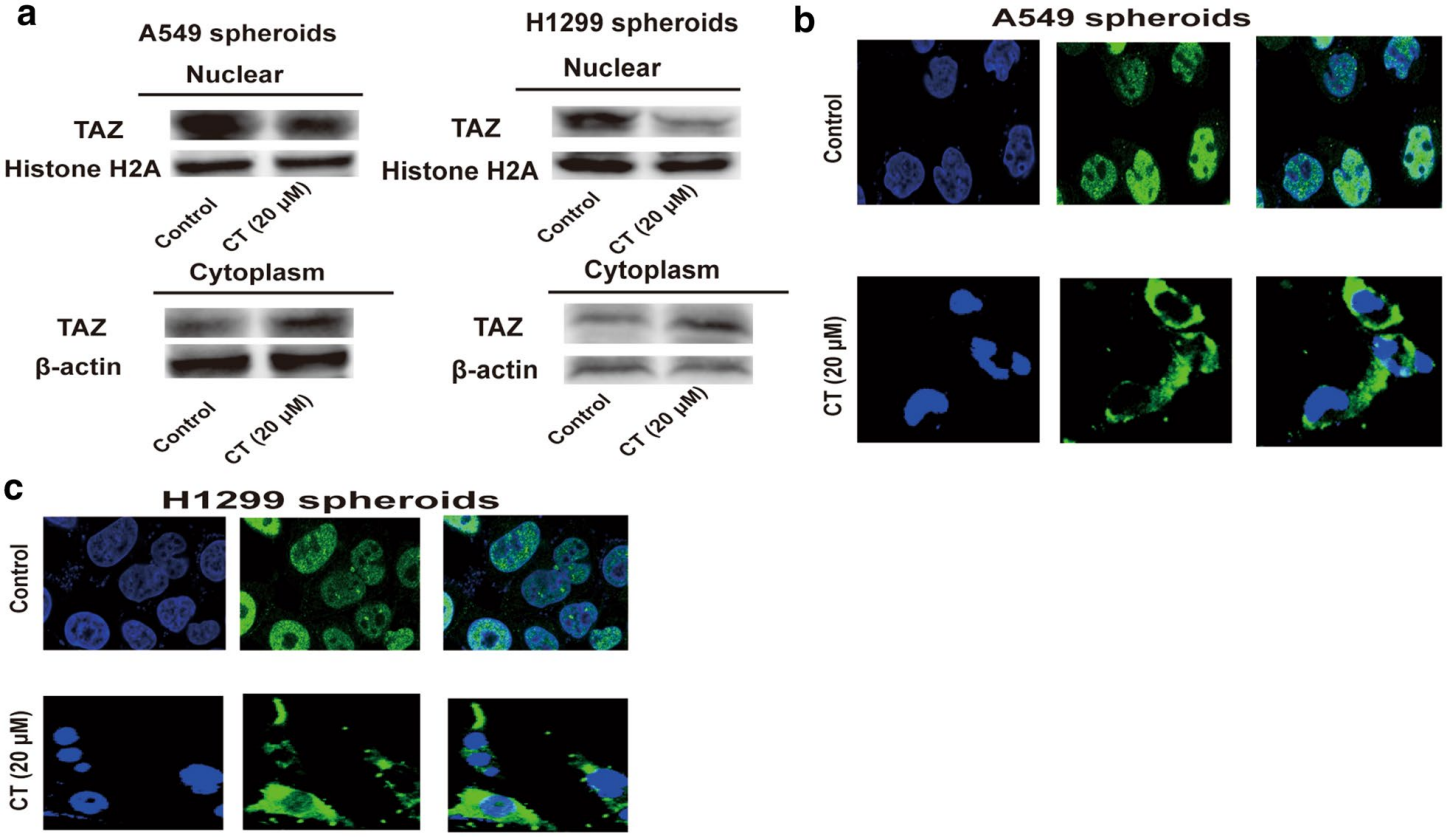
CT treatment promotes the nuclear-cytoplasm translocation in NSCLC CSCs. **a**, **b** The expression of TAZ in nuclear and cytoplasm of NSCLC spheroids was detected. **c** The location of TAZ in nuclear and cytoplasm of NSCLC spheroids was assessed via IF experiments

Then we explored the mechanisms by which CT attenuates the stemness of NSCLC CSCs. RNA-sequencing was performed in NSCLC CSCs with or without CT treatment. As shown in Fig. 4a, b, one of the mostly enriched pathways is Hippo pathway, which plays important roles in CSC expansion. However, the expression of the upstream regulators of Hippo pathway, the large tumor suppressor 1/2 (LATS1/2) was unchanged, but the downstream executor TAZ and its target genes (CTGF, TIF-1 and Smad2) expression was significantly decreased (Fig. 4b). Additionally, gene set enrichment analysis (GSEA) of this dataset revealed a positive enrichment of stem cell-differentiated signatures in NSCLC CSCs with CT treatment, and the embryonic stem cell function and adult tissue stem modules exhibited a negative enrichment (Fig. 4c–e), this effect further confirmed the inhibitory roles of CT on the stemness of NSCLC CSCs. As the expression of TAZ is regulated by its nuclear-cytoplasm translocation, we evaluated the effect of CT on the translocation from nuclear to cytoplasm of TAZ. To our surprise, it was found that the expression of TAZ in nuclear was decreased, while its expression in cytoplasm was increased (Fig. 5a, b). IF experiments obtained the consistent results (Fig. 5c). This effect means that CT promotes TAZ translocation from nuclear to cytoplasm.

### CT reduces the stemness of NSCLC CSCs dependent on TAZ expression

**Fig. 6:**
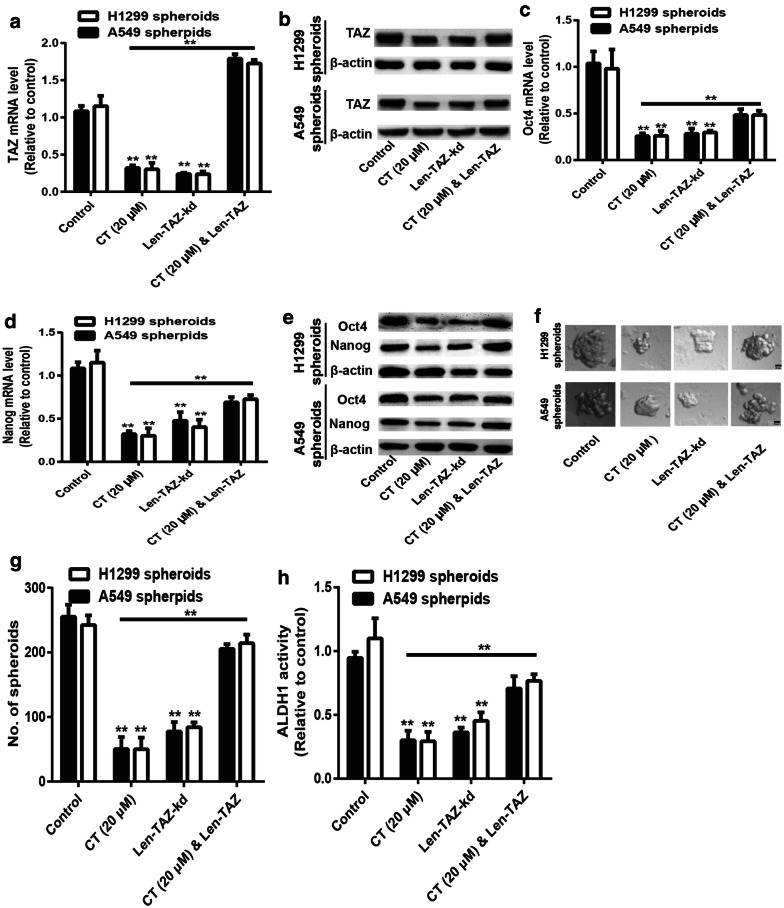
CT reduces the stemness of NSCLC CSCs dependent on TAZ expression. **a**, **b** TAZ mRNA and protein levels were examined in NSCLC spheroids with CT treatment plus TAZ overexpression or not. **c**–**e** The expression of stemness markers (Oct4 and nanog) was detected in the spheroids depicted in (**a**). **f**, **g** The capacity of spheroid formation was evaluated in the spheroids described in (**a**) via measuring spheroid size and number. **h** ALDH1 activity was determined in the spheroids described in (**a**). Data were presented as the mean ± sd, **P < 0.01 vs. control (empty lentivirus for Len-TAZ and scramble shRNA lentivirus for Len-TAZ-kd), CT (20 μM) group contained the empty lentivirus for Len-TAZ

Then, we investigated whether CT-mediated effects were dependent on TAZ expression. TAZ was overexpressed in NSCLC CSCs with CT treatment via lentivirus infection. The infection efficiency was examined (Fig. 6a, b). Additionally, overexpression of TAZ partially reversed the inhibitory effects of CT on the stemness of NSCLC CSCs, which was supported by the rescue of stemness marker expression (Fig. 6c–e), spheroid-forming ability (Fig. 6f, g) and ALDH1 activity (Fig. 6h).

### CT sensitizes NSCLC CSCs to TKI treatment and chemotherapy

**Fig. 7:**
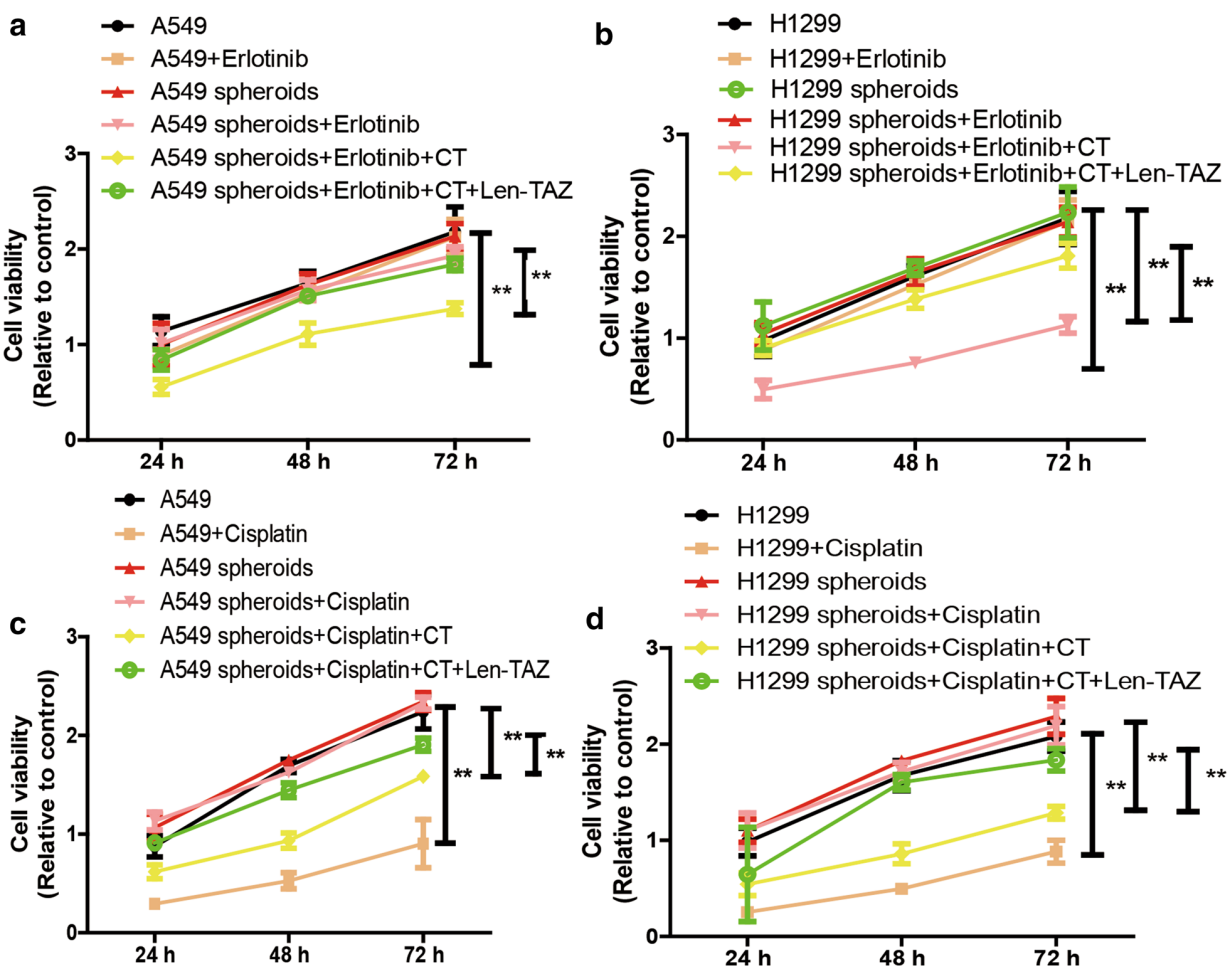
CT sensitizes NSCLC CSCs to TKI treatment and chemotherapy. **a**, **b** Cell viability was examined in NSCLC cells and spheroids treated with or without Erlotinib plus CT and TAZ overexpression or not as indicated. **c**, **d** Cell viability was detected in NSCLC cells and spheroids treated with or without Cisplatin plus CT and TAZ overexpression or not as indicated. Data were presented as the mean ± sd, **P < 0.01 vs. control (A549 or H1299)

Since CSCs are usually resistant to common drug treatment, we finally evaluated whether CT is involved in drug sensitivity of NSCLC CSCs. Because TKIs and chemotherapy are two mostly used for NSCLC treatment, Erlotinib and Cisplatin were chosen as the research subjects. As expected, NSCLC CSCs indeed exhibited an extent of resistance to Erlotinib and Cisplatin through detecting cell viability, however, CT re-sensitized NSCLC CSCs to Erlotinib and Cisplatin treatment, this effect was attenuated by TAZ overexpression (Fig. 7a–d). Therefore, our results confirm that TAZ is essential for CT-mediated inhibition on the stemness of NSCLC CSCs.

## Discussion

Our work discloses the inhibitory roles of CT in the stemness of NSCLC cells, including regulating the self-renewal capability and drug sensitivity, provoking the NSCLC CSCs entry into cell cycle. Although the previous studies have shown that CT has been proved to be an ideal inhibitor of P-gp [10] and inhibit the spheroid formation capacity and downregulate the expression of stemness genes in prostate cells and prostate CSCs [15], this study firstly reveals the CT roles in NSCLC CSC progression.

The Hippo pathway is an evolutionarily conserved signaling which play critical roles in cell differentiation and regulating organ size [22]. Alterations in Hippo pathway have been shown in regulating the stemness and targeting this pathway is correlated with CSCs progression. For example, as the stemness regulator master, sox2 maintains cancer cell stemness by antagonizing the Hippo pathway [23]. The tumor suppressor, STADR13-correlated competing endogenous RNA (ceRNA) network attenuates breast cancer cell stemness via a direct inhibition on YAP/TAZ activity (the key executors of Hippo pathway) through activating Hippo pathway [19]. MORC2 promotes the stemness and tumorigenesis by facilitating DNA methylation-dependent silencing of Hippo signaling in hepatocellular carcinoma cells [24]. Notably, as the critical executors of Hippo pathway, the WW Domain-Containing Proteins YAP and TAZ have been shown to maintain the stemness and tissue homeostasis, and facilitate tumorigenesis [25]. However, in the current study, TAZ expression but not YAP expression was reduced in NSCLC cells with CT treatment. We speculated that this different effect may be due to the different structure of YAP and TAZ, which should be explored in the future.

KRAS mutations are frequent in non-small cell lung cancer (NSCLC). However, it is difficult to targeting KRAS or the downstream/upstream effectors, such as tyrosine kinase inhibitors (TKIs). Here, NSCLC cells with KRAS mutation or not (A549 with KRAS mutation and H1299 with wild-type KRAS) were chosen as the research subjects. Our results demonstrate that CT attenuated the stemness of the KRAS-mutant and –wild-type NSCLC cells, implying CT has no selectivity on NSCLC cells. Notably, although PI3K/Akt pathway has been shown to promote tumor progression [26, 27] and Akt and PI3K inhibitors are in clinical trials [28], most of them failed in NSCLC and resistance always happens. The previous work indicates that PI3K/Akt signaling is suppressed in quiescent acute myeloid leukemia stem cells [29] and inhibition of AKT activity increased the ratio of the G_0_-phased cells [30]. And limonin attenuates the stemness of hepatocellular carcinoma cells by reducing cellular quiescence through activating PI3K/Akt signaling [17]. These results suggest that PI3K/Akt activity was different in cells with or without stemness. As shown in Fig. 4 results, PI3K/Akt pathway was activated in NSCLC CSCs with CT treatment, and overexpression of TAZ just partially reversed the inhibitory effects of CT (Fig. 5), demonstrating that CT may attenuate the stemness of NSCLC CSCs through other pathways, such as PI3K/Akt. In addition, in vivo experiments should be constructed to confirm the conclusion. Importantly, Salvia miltiorrhiza has the highest application frequency in the compatibility of traditional Chinese medicine for promoting blood circulation and removing blood stasis and the other main components of Salvia miltiorrhiza, such as tanshinone I, dihydrotanshinone and tanshinone IIA, have been confirmed to be involved in CSC progression [10–15]. Combined with these previous results, this study attempts to use modern medical mechanism to study and explain the theory of traditional Chinese medicine, reveal the potential mechanism of blood-activiating and stasis-dissolving drugs to resist chemoresistance of breast cancer. We strongly believe that Salvia miltiorrhiza, which is a typical blood-activiating and stasis-dissolving drug and has been proved to be used in clinic, may be used to target CSCs, this could be explored in the future.

## Conclusions

All in all, our results indicate that CT reduces NSCLC CSC stemness, providing a potential drug for combinatory using for NSCLC treatment.

## Supplementary information

**Additional file 1: Figure S1.** The images of the original western blots in triplicate.

## Data Availability

All data generated or analysed during this study are included in this published article.

## Abbreviations

CSCs: Cancer stem cells
NSCLC: Non-small cell lung cancer
CT: Cryptotanshinone
qRT-PCR: Quantitative real-time PCR
TKI: Tyrosine kinase inhibitor
STR: Short tandem repeat
EdU: 5-Ethynyl-2′-deoxyuridine
ALDH: Acetaldehyde dehydrogenase
LATS1/2: Large tumor suppressor 1/2
GSEA: Gene set enrichment analysis
ceRNA: Competing endogenous RNA
