# Supplementary figures and images for: Cryptotanshinone attenuates the stemness of non-small cell lung cancer cells via promoting TAZ translocation from nuclear to cytoplasm
Source: Chin Med. 2020 Jun 30;15:66. doi: 10.1186/s13020-020-00348-4 (PMC7325009; doi:10.1186/s13020-020-00348-4)

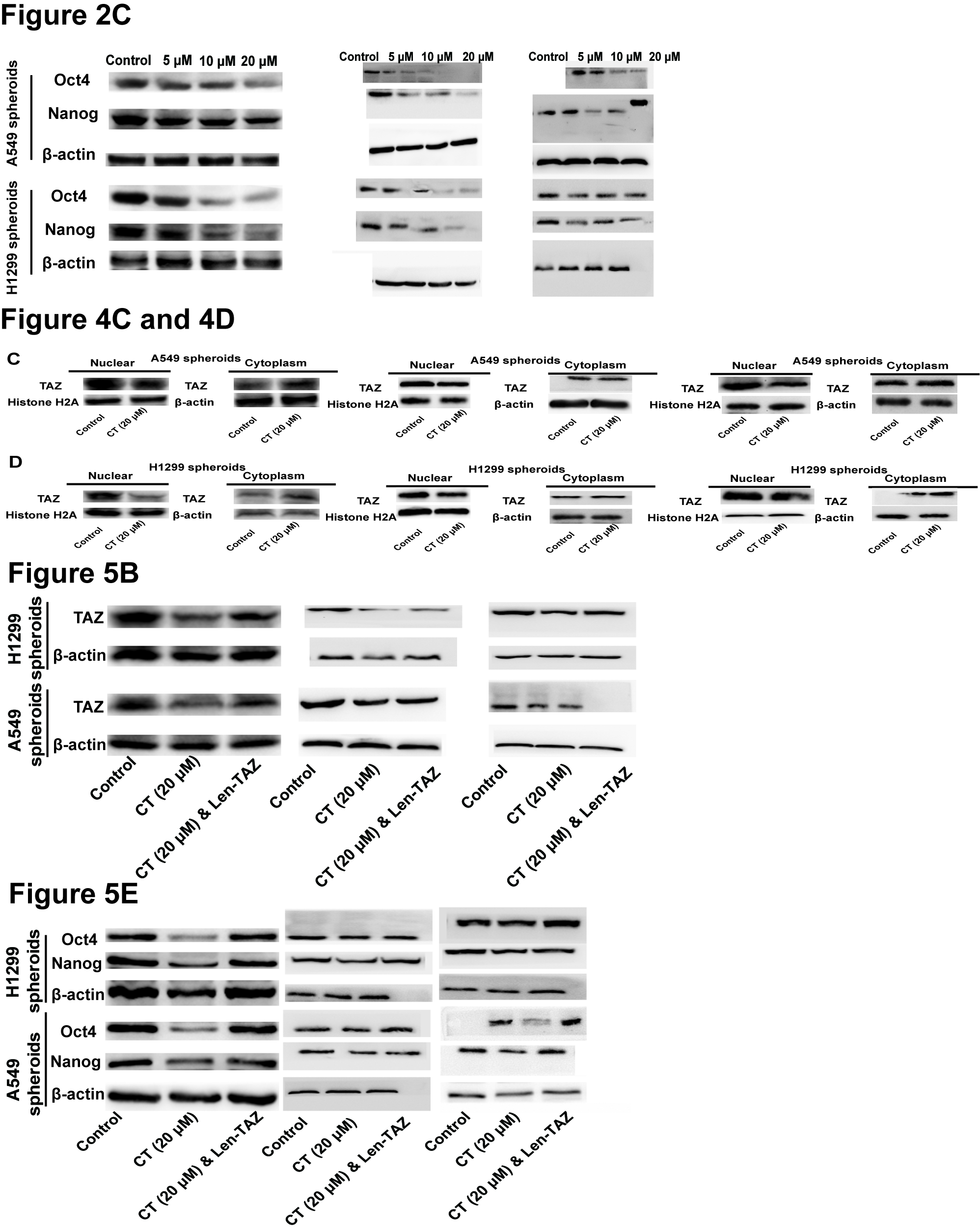

Supplement: Supplementary file 1 — Additional file 1: Figure S1. The images of the original western blots in triplicate. [file 13020_2020_348_MOESM1_ESM.tif]
